# Draft Genome Sequences of 12 Lactobacillus reuteri Strains of Rodent Origin

**DOI:** 10.1128/MRA.00004-20

**Published:** 2020-02-13

**Authors:** Shenwei Zhang, Mustafa Özçam, Jan-Peter van Pijkeren

**Affiliations:** a Department of Food Science, University of Wisconsin—Madison, Madison, Wisconsin, USA; University of Maryland School of Medicine

## Abstract

Lactobacillus reuteri is a bacterial gut symbiont found in many vertebrate animals. The genetic heterogeneity of L. reuteri is likely to contribute to differences in ecological performance within a host. Here, we report the draft genome sequences of 12 L. reuteri strains of rodent origin.

## ANNOUNCEMENT

Lactobacillus reuteri is a Gram-positive bacterium that can be found in the digestive tract of many vertebrate animals, including humans, pigs, rodents, sheep, cattle, and birds (1). In rodents, L. reuteri resides in the forestomach and is one of the most abundant species (2). Previous studies revealed that some L. reuteri strains are more competitive than others in the rodent gastrointestinal tract (3, 4); however, little is known about the molecular mechanisms that drive gut fitness. As a first step toward understanding the mechanisms that contribute to gut fitness, we sequenced 12 L. reuteri rodent isolates, some of which displayed different fitness traits (4).

Initial identification of the L. reuteri strains described in this study was previously determined by 16S rRNA sequence analysis (4), with the exception of Lr4000, for which we do not know the original identification method. From a glycerol stock, each of the 12 L. reuteri isolates of rodent origin was inoculated in De Man-Rogosa-Sharpe (MRS) medium (Difco, BD Biosciences) and incubated for 12 h at 37°C. The cultures were grown on MRS agar, and we transferred a single colony into MRS broth, which was incubated for 12 h at 37°C. Genomic DNA was isolated and purified with the Wizard genomic purification kit (Promega), and purified DNA was quantified with the Qubit double-stranded DNA (dsDNA) high-sensitivity (HS) assay kit (Life Technologies). DNA was submitted to the University of Wisconsin—Madison Biotechnology Center. Samples were prepared using a TruSeq Nano DNA low-throughput (LT) library prep kit (Illumina, Inc.). The finished libraries were assessed using an Agilent Bioanalyzer and a Qubit dsDNA HS assay kit to determine the quality and quantity of the samples, respectively. DNA libraries were standardized to 2 nM. Paired-end (250-bp) sequencing was performed using an Illumina MiSeq sequencer and a MiSeq 500-bp v2 sequencing cartridge. Images were analyzed using the standard Illumina Pipeline v1.8.2. The total numbers of raw reads and bases are displayed in Table 1. Adaptor sequences in the reads were trimmed with fastp v0.20.0 using default settings (5). *De novo* assembly of sequence reads was performed with SPAdes v3.13.1 with the default k values equal to 21, 33, 55, 77, 99, and 127 (6). Contigs that are smaller than 200 bp were removed using the BBMap suite v38.67 reformat.sh function with the “minlength=200” option (7). We annotated the genomes with the NCBI Prokaryotic Genome Annotation Pipeline (8). The summary statistics of the genomes of the 12 strains are shown in Table 1.

**TABLE 1:** Genome sequencing and assembly statistics

Strain	Host	Total no. of reads	Total no. of bases	Coverage (×)	No. of contigs	*N*_50_ (bp)	Genome length (bp)	GC content (%)	GenBank accession no.	SRA accession no.
100-93	Mouse	1,970,280	989,080,560	197	141	49,708	2,060,228	38.5	WJMU00000000	SRR10435990
6799jm-1	Mouse	1,502,159	754,083,818	149	130	46,161	2,145,408	38.5	WJMV00000000	SRR10435989
AD23	Rat	1,612,412	754,083,818	164	250	38,503	2,041,907	38.5	WJMW00000000	SRR10435986
CR	Rat	917,758	460,714,516	93	64	126,392	2,172,995	38.5	WJMX00000000	SRR10435985
L1600-1	Mouse	1,169,340	587,008,680	118	80	64,150	2,066,036	38.6	WJMY00000000	SRR10435984
L1604-1	Mouse	1,336,692	671,019,384	138	62	288,224	2,120,830	38.3	WJMZ00000000	SRR10435983
Lr4020	Mouse	807,917	405,574,334	66	105	80,427	2,255,929	38.3	WJNA00000000	SRR10435982
Lr4000	Mouse	1,289,342	647,249,684	116	70	279,211	2,396,776	38.6	WJNB00000000	SRR10435981
N2J	Rat	1,294,080	649,628,160	121	142	106,800	2,114,585	38.5	WJNC00000000	SRR10435980
N4I	Rat	1,509,197	757,616,894	148	111	145,434	2,089,201	38.6	WJND00000000	SRR10435979
Rat19	Rat	1,134,426	569,481,852	118	98	69,801	2,101,955	38.6	WJNE00000000	SRR10435988
One-one	Rat	1,322,734	664,012,468	133	68	120,479	2,158,620	38.6	WJNF00000000	SRR10435987

### Data availability.

The genome sequences of these 12 L. reuteri strains were deposited in DDBJ/ENA/GenBank, and all raw sequence data are publicly available in the Sequence Read Archive (SRA). GenBank and SRA accession numbers are listed in Table 1.
